# Evidence of exceptional oyster‐reef resilience to fluctuations in sea level

**DOI:** 10.1002/ece3.3473

**Published:** 2017-10-31

**Authors:** Justin T. Ridge, Antonio B. Rodriguez, F. Joel Fodrie

**Affiliations:** ^1^ Institute of Marine Sciences University of North Carolina at Chapel Hill Morehead City NC USA

**Keywords:** accretion, coastal resilience, *Crassostrea virginica*, estuarine, green infrastructure, oyster reefs, sea level, surface elevation

## Abstract

Ecosystems at the land–sea interface are vulnerable to rising sea level. Intertidal habitats must maintain their surface elevations with respect to sea level to persist via vertical growth or landward retreat, but projected rates of sea‐level rise may exceed the accretion rates of many biogenic habitats. While considerable attention is focused on climate change over centennial timescales, relative sea level also fluctuates dramatically (10–30 cm) over month‐to‐year timescales due to interacting oceanic and atmospheric processes. To assess the response of oyster‐reef (*Crassostrea virginica*) growth to interannual variations in mean sea level (MSL) and improve long‐term forecasts of reef response to rising seas, we monitored the morphology of constructed and natural intertidal reefs over 5 years using terrestrial lidar. Timing of reef scans created distinct periods of high and low relative water level for decade‐old reefs (*n* = 3) constructed in 1997 and 2000, young reefs (*n* = 11) constructed in 2011 and one natural reef (approximately 100 years old). Changes in surface elevation were related to MSL trends. Decade‐old reefs achieved 2 cm/year growth, which occurred along higher elevations when MSL increased. Young reefs experienced peak growth (6.7 cm/year) at a lower elevation that coincided with a drop in MSL. The natural reef exhibited considerable loss during the low MSL of the first time step but grew substantially during higher MSL through the second time step, with growth peaking (4.3 cm/year) at MSL, reoccupying the elevations previously lost. Oyster reefs appear to be in dynamic equilibrium with short‐term (month‐to‐year) fluctuations in sea level, evidencing notable resilience to future changes to sea level that surpasses other coastal biogenic habitat types. These growth patterns support the presence of a previously defined optimal growth zone that shifts correspondingly with changes in MSL, which can help guide oyster‐reef conservation and restoration.

## 1 INTRODUCTION

Climate change poses a significant threat to ecosystems across the globe with pronounced impacts to biogeography, manifesting most prominently at the edges of species ranges, near an organism's threshold tolerance to physicochemical or biotic controls. Changes to the environment along these boundaries could result in a variety of outcomes including species adaptations (Hoffmann & Sgro, 2011), changes to phenology (Edwards & Richardson, 2004; Poloczanska et al., 2013), range shifts (Chen, Hill, Ohlemüller, Roy, & Thomas, 2011; Davis & Shaw, 2001; Poloczanska et al., 2013), community and trophic restructuring (Edwards & Richardson, 2004; Walther et al., 2002), and even localized extinction (Colwell, Brehm, Cardelus, Gilman, & Longino, 2008; Pinsky, Worm, Fogarty, Sarmiento, & Levin, 2013). The magnitude of these responses will depend on an organism's sensitivity to the suite of environmental factors that may be undergoing change or the resultant altered biotic relationships, the rate at which the system is changing (Ackerly et al., 2010), and the reaction time of the species to adapt.

The response and reaction time of various organisms to climate fluctuations are highly specific among different taxa. Conditions detrimental to fitness may occur if there is a notable lag in community response to climate alterations, as seen with forest communities and temperature (Bertrand et al., 2011), or a species unable to shift correspondingly to the vector and acceleration at which an environmental variable, such as temperature or average rainfall, is changing (Burrows et al., 2014; Dobrowski et al., 2013; Zhu, Woodall, & Clark, 2012). The nature of environmental shifts across geographic space means mobile organisms can respond more readily by migrating (Pinsky et al., 2013), whereas sessile organisms must rely on adaptation, propagation, and habitat modification to maintain their populations (Bertrand et al., 2011). As many communities depend on the persistence of habitat‐forming foundation species, it is crucial that these sessile ecosystem engineers keep pace with climate changes to sustain habitat area and quality (Colwell et al., 2008; Kirwan & Megonigal, 2013; Ridge et al., 2015).

Biogenic habitats are experiencing environmental change in a variety of forms, including temperature, precipitation/desertification, ocean acidification, and sea‐level rise (SLR); all of which vary in rate geographically and can interact to cause complex responses in ecological communities as populations react differently (Tingley, Koo, Moritz, Rush, & Beissinger, 2012). While many of these climatic factors shift laterally across a geographic space, SLR also presents change in the vertical, which is particularly important for developed coastal areas where infrastructure prevents upland migration. Intertidal and shallow subtidal biogenic habitats exist in a narrow elevation range due to a combination of biophysical intolerance and interspecific interactions (Bertness & Ellison, 1987; Fodrie et al., 2014; Paine, 1971). Fluctuations in sea level can represent a dramatic change to species that are relegated to intertidal zones, such as saltmarshes and mangroves, because it changes the inundation time during a tidal cycle. The change in sea level may be significant compared to the overall range of elevations the organisms occupy. If these foundation species cannot maintain their surface elevations compared to relative SLR (RSLR, the combination of eustatic sea‐level rise and local shifts to continental crust), they will become imperiled by the stress of saltwater submergence, which could result in a loss of their supported communities and associated ecosystem services (Kirwan & Megonigal, 2013; Lovelock et al., 2015).

While sea level along the coast of the United States is generally rising 2–6 mm/year, it fluctuates significantly from year to year, seasonally, and even on shorter timescales (weeks to months). These changes can range from 15 to 20 cm interannually with the most dramatic being greater than 30 cm (Morris et al., 2002; Sweet, Zervas, & Gill, 2009; Sea Level Trends, NOAA Tides & Currents). Some of this variation is due to seasonal temperature and wind climate, but pronounced deviations may also arise with the complex interconnectivity of the North Atlantic Oscillations (NAO), prolonged or frequent storm activity, and sea‐level anomalies linked to the strength of the Gulf Stream (Ezer, 2016; Ezer, Atkinson, Corlett, & Blanco, 2013; Goddard et al.*,* 2015; Kolker & Hameed, 2007; Sweet, Zervas, & Gill, 2009). Losada et al. (2013) demonstrated that interannual shifts in sea level in other areas of the Atlantic Ocean can be on the order of 4–12 cm, with ENSO‐induced sea‐level shifts exceeding historical RSLR and an increased frequency in sea‐level extremes occurring in recent decades. Short‐term elevations in sea level are responsible for more frequent flooding along the U.S. East Coast (Ezer & Atkinson, 2014) and increased coastal erosion (Theuerkauf, Rodriguez, Fegley, & Luettich, 2014). These fluctuations in sea level may have a marked impact on coastal and estuarine habitats as their regularity and longevity are expected to increase (Ezer & Atkinson, 2014).

The persistence of biogenic habitats, along with the critical services they provide to ecosystems and coastal infrastructure, is uncertain in the face of accelerated RSLR. Vegetated habitats (saltmarshes, mangroves, and seagrasses) alter their surface elevations through passive trapping of sediment from the water column, accumulation of annual aboveground biomass, and by augmenting belowground biomass forcing the sediment surface upwards (Morris et al.*,* 2002). Habitats constructed by invertebrates (e.g., coral reefs, oyster reefs, worm reefs) rely on individual growth and gregarious settlement to maintain their placement in suitable conditions, with multiple generations building on one another. Given the range of accretion rates exhibited by these ecosystem engineers (Baustian, Mendelssohn, & Hester, 2012; Bhomia, Inglett, & Reddy, 2015; Cahoon et al.,^2006^; Perry et al., 2015; Sasmito, Murdiyarso, Friess, & Kurnianto, 2016), many will maintain their relative position with moderate rates of RSLR, while higher rates of RSLR may result in massive loss of coastal habitats along large geographic stretches due to drowning and compression against coastal infrastructure (Pontee, 2013).

**Figure 1:**
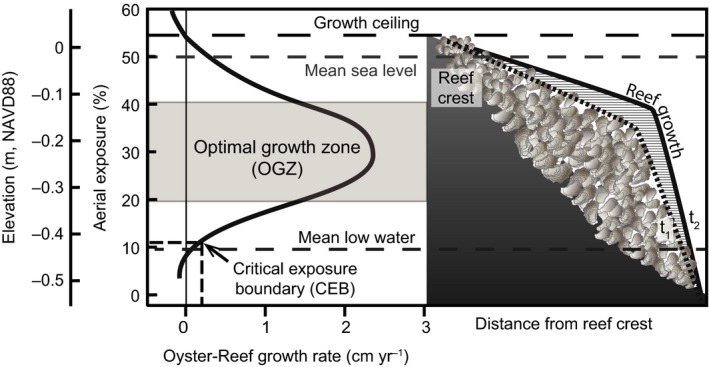
Reef growth conceptual model adapted from Ridge et al. (2015) that predicts oyster‐reef growth rate with aerial (tidal) exposure. Relevant elevations in NAVD88 are provided for aerial exposures (%) for the Cape Lookout region of North Carolina. The lower critical exposure boundary occurs where oyster‐reef growth equals the rate of relative SLR (RSLR), shifting correspondingly as RSLR changes. Oyster‐reef growth is illustrated (right panel) across a hypothetical reef‐elevation profile using dotted (time 1) and solid (time 2) profile lines

Oyster reefs are ubiquitous features within temperate and subtropical estuaries, spanning from the intertidal to subtidal zones depending on salinity and climate (Baggett et al., 2015; Walles et al., 2016). While oysters provide many important benefits to the ecosystem, populations are recovering from decimation during the last century (Beck et al., 2011). Previous work examining intertidal oyster‐reef growth indicates that constructed *Crassostrea virginica* reefs have a relatively high growth capacity compared to other coastal habitats (Rodriguez et al., 2014), far outpacing any predicted rate of RSLR. However, growth rates are highly variable across reef‐elevation gradients due to stress associated with exposure (desiccation) and submergence (competition and predation), with the reef crest and base exhibiting stunted or lack of growth (critical exposure boundaries) and the sides growing at the highest rate (optimal growth zone [OGZ], Ridge et al., 2015) (Figure 1). In the lower portions of estuaries, where salinities are typically greater than 30 ppt, *C. virginica* reefs cannot persist in the subtidal zone due to overwhelming predation and competition by species that are intolerant to exposure (Fodrie et al., 2014; Powers et al., 2009), indicating that transitioning from intertidal to subtidal conditions will place reefs that cannot keep pace with rising seas in peril (Ridge et al., 2015). While oyster‐reef growth patterns are well constrained over decadal scales, their sensitivity to changing sea levels over monthly to yearly timeframes is still relatively unknown. Considering the degree to which sea level can fluctuate from weeks to months, understanding how the critical boundaries and OGZ will shift in response is necessary information for proper timing and siting of oyster restoration projects as well as assessing how future trends of RSLR will affect reef persistence, which in many estuaries is the only available hard substrate.

## 2 MATERIALS AND METHODS

### 2.1 Study area

**Figure 2:**
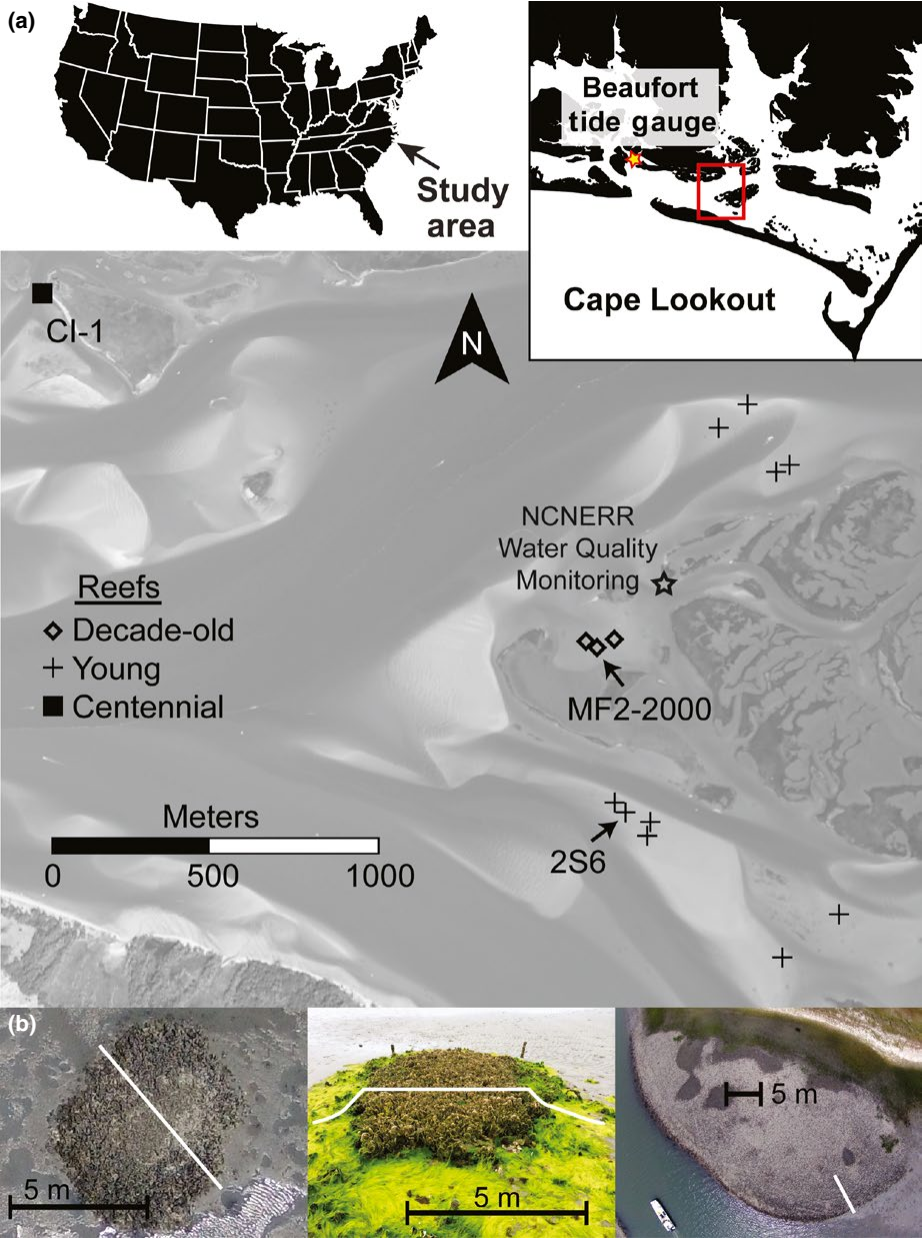
(a) Study area map of Back Sound, North Carolina. All reefs are located within the Rachel Carson Research Reserve (NC Coastal Reserves/National Estuarine Research Reserves) in Beaufort, NC. (b) Examples of the three generations of reefs samples, decade‐old (left, constructed in 1997 and 2000), young (middle, constructed in 2011), and centennial (right, natural). White bars represent transects for elevation profiles presented in Figure 5. Black bars are included for scale

This study was conducted using *C. virginica* oyster reefs located in the Rachel Carson Research Reserve (North Carolina National Estuarine Research Reserve, NCNERR), Back Sound, North Carolina, USA (all reefs are within 2 km of 34.693007°N, 76.621709°W, see Figure 2a). The area is comprised of channelized sandflats and marsh islands (*Spartina alterniflora*). Tides are semidiurnal with a mean range of 0.9 m and mean sea level at −0.03 m (all elevations reported in the North American Vertical Datum of 1988 [NAVD88]). Oyster reefs are predominantly intertidal in areas around Back Sound, occurring along marsh shorelines (fringing) or isolated on sandflats (patch).

### 2.2 Study design

**Figure 3:**
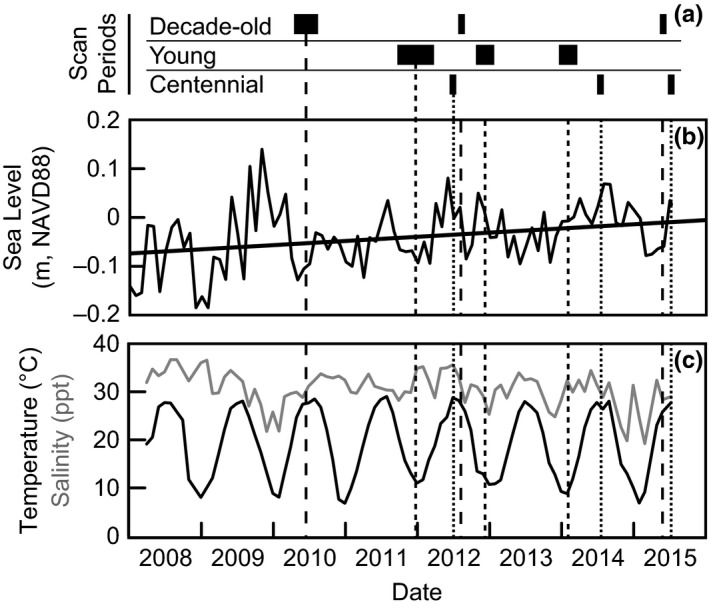
(a) Timeline of reef scans obtained for each reef generation. Width of bars indicates the length of time to obtain scans during each sampling period. Each reef generation scanning period is denoted by a unique dashed line through the rest of the figure. See Table 1 for additional information. (b) Monthly mean sea level data from the NOAA Tide Gauge in Beaufort (Station ID 8656483). Data are reported as elevations (m NAVD88), and the linear trend of the sea level data is plotted. (c) Mean monthly water temperature (°C, black line) and salinity (ppt, gray line) obtained at the Rachel Carson Research Reserve (NCNERR)

Our study included an assortment of reefs of different ages (grouped into three “generations”) ranging from 2 years to a century old (Figure 2b). Constructed reefs ranged from 5 to 10 m in diameter, similar in size to many natural reefs in our study area, while the natural reef included in this study was one of the larger reefs locally, approximately 15 × 50 m (width × length). Constructed reefs began as mounds of loose, recycled oyster shell (cultch shell) measuring 3 × 5 × 0.15 m (width × length × height), followed by natural recruitment of oyster larvae from the estuary. Growth (cm/year) of three reefs constructed over a decade prior (Grabowski, Hughes, Kimbro, & Dolan, 2005; hereafter “decade‐old reefs”), including one reef constructed in 1997 and two in 2000, was measured from Spring 2010 to Spring 2012, and from Spring 2012 to Spring 2015 (Table 1, Figure 3a). Growth of eleven reefs constructed in 2011 (hereafter “young reefs”), and scanned each subsequent winter was measured over nearly a 1‐year period in 2012 and nearly a 1‐year period in 2013 (Table 1, Figure 3a). Finally, one natural reef nearly 100 years old (based on old nautical maps, hereafter “centennial reef”) was initially scanned in 2012 and growth was calculated from 2012 to 2014 and from 2014 to 2015 (Table 1, Figure 3a).

**Table 1:** Reef name sorted by type, average area scanned for each reef type, and date of each terrestrial laser scan mapping

Reef name	Mean area (m^2^)	Scan 1	Scan 2	Scan 3
Decade‐old	52.8			
MF2‐1997		June 2010	July 2012	May 2015
MF1‐2000		June 2010	July 2012	May 2015
MF2‐2000		April 2010	July 2012	May 2015
Young	39.1			
1L5		December 2011	December 2012	February 2014
1L6		December 2011	January 2013	March 2014
1S5		December 2011	January 2013	March 2014
1S6		December 2011	December 2012	February 2014
2L5		October 2011	January 2013	January 2014
2L6		March 2012	January 2013	January 2014
2S5		—	January 2013	January 2014
2S6		March 2012	January 2013	January 2014
3L5		October 2011	January 2013	February 2014
3S5		October 2011	January 2013	February 2014
4S5		January 2012	January 2013	March 2014
Centennial	222.5			
CI‐1		June 2012	June 2014	June 2015

**Figure 4:**
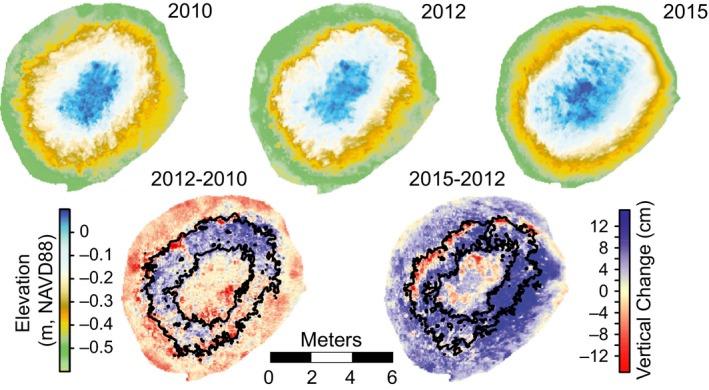
Digital elevation models and subsequent subtraction maps from the decade‐old reef MF2‐2000. The reef was mapped with terrestrial lidar in 2010 (April), 2012 (July), and 2015 (May). Black contours on the subtraction maps represent the boundaries of the optimal growth zone (OGZ) (20%–40% aerial exposure) referencing elevations from 2010 to 2012 (initial scans), respectively

To examine fine‐scale growth across oyster reefs, terrestrial lidar (Riegl LMSZ210ii laser scanner) was used to image reefs (Figure 4). Terrestrial laser scanning followed previously reported methods (Rodriguez et al., 2014), using RTK‐GPS‐positioned reflectors to georectify the point cloud to less than 1‐cm horizontal and 1.5‐cm vertical accuracy. Elevations were recorded in the North American Vertical Datum of 1988 (NAVD88). Reef‐mapping with lidar required dry weather and a low spring tide, providing only a narrow operating window to scan a reef, which typically took an hour. As such, it sometimes required several days to several months to acquire all the scans of each reef generation, particularly in the case of the young reefs, which were numerous and widely separated (denoted by the bar widths in Figure 3a). Within each reef generation, we collected scans during the same season and normalized the data to annual rates to avoid uneven seasonal influences across the time steps. The combination of RiSCAN Pro (Riegl) and Merrick Advanced Remote Sensing (MARS 7.1) software packages was used to extract ground points, which were then gridded (5‐cm grid spacing) in Surfer 13 (Golden Software) using the Kriging algorithm to create digital elevation models (DEMs). Consecutive DEMs were subtracted, and the resulting elevation change was linked to the corresponding grid‐cell elevation from the initial DEM and those linked data were binned to determine average vertical change at 2‐cm elevation bins across a reef (Figure 4). Total volume change for each elevation bin was then calculated by multiplying the grid‐cell area (25 cm^2^) by the total number of grid cells and average vertical change within a particular elevation bin. A portion of the centennial reef was excluded from the growth analysis due to signs of heavy disturbance from harvesting and boat impacts along the adjacent tidal channel.

Fluctuations in sea level from 2009 to 2015 were examined using monthly mean sea‐level (MSL) data from a NOAA tide gauge (NOAA Tides & Currents Station ID: 8656483, Beaufort, North Carolina) located approximately 5 km northeast of the reefs. We used VDatum 3.6 (NOAA/NOS Vertical Datum Transformation) to transform monthly MSL data into elevations in m NAVD88. Average sea level during scan periods was then calculated for each time step. Six‐minute water level data were also queried from the Beaufort tide gauge and used to construct elevation‐exposure histograms to predict the OGZ elevation range for each scan comparison. Local water salinity and temperature recorded in Back Sound by the North Carolina Coastal Reserves (North Carolina National Estuarine Research Reserve) during the study period were also examined by month to elucidate if patterns in reef growth were tied to changes in water quality. Using JMP v12 (SAS Institute Inc.), regression analyses were run on monthly mean water temperatures and salinity during the overall scan period (June 2010–June 2015) to determine any trends in water quality beyond regular seasonal fluctuations. Additionally, data were transformed (cube transformation) to meet assumptions for parametric analysis, and a series of *t*‐tests were run to compare monthly mean water temperatures and salinities between scan periods within each reef generation.

## 3 RESULTS

### 3.1 Water level and quality

Monthly MSL data from 2009 to 2015 indicate that sea level in the study area was −0.028 ± 0.062 m NAVD88 (mean ± *SD*) (Figure 3b). Prior to the start of scanning, the study area experienced prolonged levels of high water from frequent sea‐level anomalies during the fall and winter of 2009–2010 that persisted for 5 months (Theuerkauf et al., 2014). The sea‐level peak in 2009 corresponds to the November Mid‐Atlantic nor'easter that spawned from the remnants of Hurricane Ida (deemed Nor'Ida). This peak in sea level was followed by relatively low water during 2010 and 2011. Prolonged periods of elevated monthly sea levels (above mean longer than three consecutive months) occurred during 2012, 2014, and 2015 (Figure 3b, Table 2).

**Table 2:** Summary of peak growth, sea level, temperature, and salinity for each time step by reef generation

Reef type	Time step	Elevation of growth peak (cm)^a^	Mean sea level (cm)^a^	Mean temperature (°C)	Mean salinity (ppt)	Peak growth (cm/year)	Δ Growth peak elevation (cm)	Δ Sea level (cm)
Decade‐old	1	−17	−5.3	19.7	31.8	1.90	+8.0	+3.7
	2	−9	−1.6	18.6	29.6^b^	1.72		
Young	1	−23	−1.7	19.7	32.4	3.68	−2.0	−2.6
	2	−25	−4.3	18.2	29.7^b^	6.67		
Centennial	1	−29	−2.1	18.8	30.6	2.89	+30.0	+3.9
	2	1	1.8	18.5	27.7	4.36		

The elevation change in growth peak occurrence and sea level between each periods is also included.

a Elevations reference North American Vertical Datum of 1988.

b Significant difference (*p < *.05) from previous time step.

Water temperature consistently fluctuated seasonally during the study period (Figure 3c), and mean monthly temperatures ranged from 5 to 30°C with no significant trend during the study period (*R* ^2^ = −0.02, *F*_1,57_ = 0.99, *p* = .33). Furthermore, all comparisons of water temperatures between scan periods within reef generations did not yield significant differences (Tables 2 and S1). Overall, salinity declined during the study period (*R* ^2^ = 0.17, *F*_1,56_ = 11.1, *p* = .0015). Prior to the first scan period, the area experienced a distinct drop in salinity with high water associated with Nor'Ida (winter 2009–2010). Subsequent drops in salinity occurred periodically during the scan periods, with the most pronounced declines coinciding with high water in late 2014 and low water in early 2015 (Figure 3c). Comparisons of salinity between scan periods indicated significant decreases in salinity across time steps in both decade‐old and young reefs, but only marginal significance (if α = 0.1) in the centennial reef (Tables 2 and S1).

### 3.2 Reef growth

**Figure 5:**
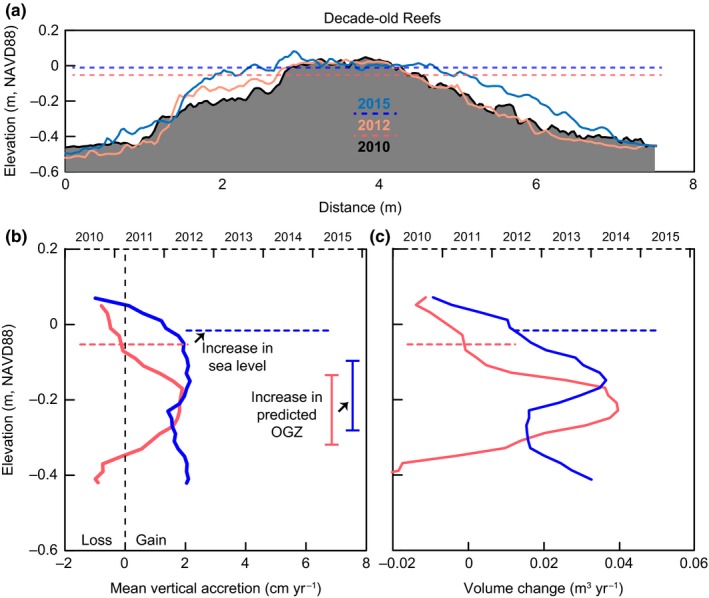
Decade‐old reef reefs constructed in 1997 (*n* = 1) and 2000 (*n* = 2) scanned in 2010, 2012, and 2015. (a) Vertical profiles of MF2‐2000 during each scan with average sea levels between each scan period (dashed lines). (b) Mean vertical accretion rates by elevation (solid lines) and mean relative sea levels (dashed horizontal lines) during the scan time steps. Light red and dark blue bracketed bars represent the predicted optimal growth zone (OGZ) based on water level data for relevant scan periods. (c) Reef volume change at each elevation (solid lines) with mean sea level data (dashed lines)

For the decade‐old reefs, average sea level between the two periods increased approximately 4 cm from −0.053 m NAVD88 (averaged through Spring 2010–Spring 2012) to −0.016 m NAVD88 (average through Spring 2012–Spring 2015) (Figure 5a,b), with prolonged higher water during the warm seasons (April through September) of 2012 (0.004 m NAVD88) and 2014 (0.032 m NAVD88). Initial elevations (2010–2012) of the OGZ on decade‐old reefs were positioned between −0.15 and −0.30 m NAVD88 with growth peaking at 2 cm/year (Figure 5b, Table 2). From 2012 to 2015, the maximum growth rate on these reefs remained at 2 cm/year, but the zone of high growth expanded 34%, spanning the elevations from −0.05 to −0.49 m NAVD88. The upper critical no‐growth boundary, or growth ceiling, increased in elevation from −0.07 m to 0.05 m NAVD88, while the lower critical exposure boundary was not evident in the second time step, indicating growth over the entire reef surface. Greatest volume increases occurred within the OGZ during both time steps, yielding an average of nearly 0.04 m^3^/year (Figure 5c). Minor reef volume loss occurred at the reef crests during both time periods at nearly −0.01 m^3^/year. The base of the decade‐old reefs lost volume (−0.02 m^3^/year) during the first time step, but regained it over the second (0.03 m^3^/year).

**Figure 6:**
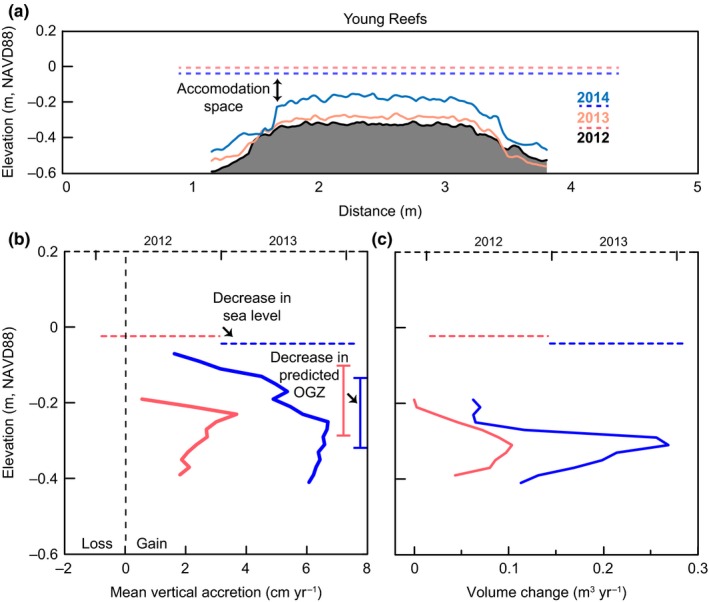
Young reefs constructed in 2011 (*n* = 10) measured at the beginning of 2012, 2013, and 2014. (a) Vertical profiles of reef 2S6 during each scan with average sea levels between each scan period (dashed lines). (b) Mean vertical accretion rates by elevation (solid lines) and mean relative sea levels (dashed horizontal lines) during the scan time steps. Light red and dark blue bracketed bars represent the predicted optimal growth zone (OGZ) based on sea‐level data for relevant scan periods. (c) Reef volume change at each elevation (solid lines) with mean sea‐ level data (dashed lines)

Mean annual sea level during the 2012 and 2013 scan periods for the young reefs dropped 2.6 cm, from −0.017 m NAVD88 (averaged through Winter 2012–Winter 2013) to −0.043 m NAVD88 (averaged through Winter 2013–Winter 2014) (Figure 6a). Over the course of 2012, young reefs experienced a maximum growth rate of 3.6 cm/year at −0.23 m NAVD88 (Figure 6b, Table 2). The following year, the OGZ shifted to a lower elevation with greatest growth (6.7 cm/year) at −0.25 m NAVD88. These young reefs lack both the lower and upper critical exposure boundaries because they occupy shallower substrates than the decade‐old reefs and have also not yet grown to sea level (i.e., filling the accommodation space). In terms of volume change, the young reefs accumulated the most volume at the base of the OGZ during each time step, increasing from 0.1 m^3^/year to almost 0.3 m^3^/year between the two time periods, a magnitude greater volume gain than the decade‐old reefs (Figure 6c).

**Figure 7:**
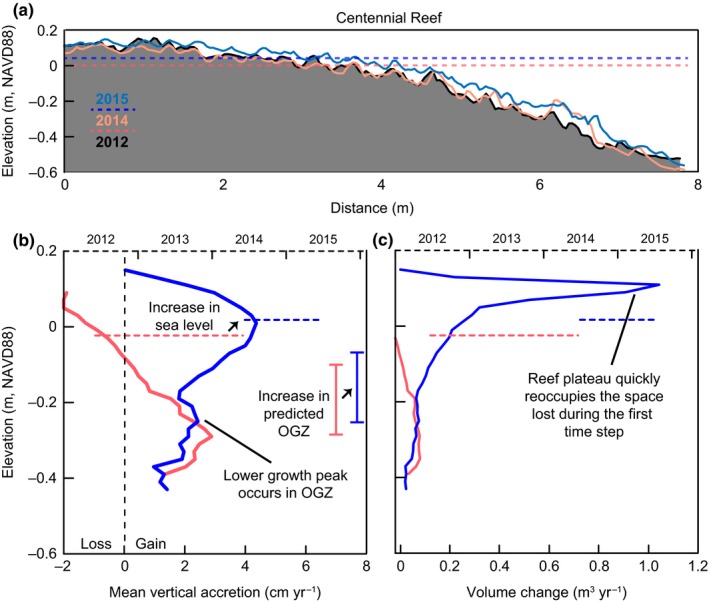
The natural, centennial reef scanned in 2012, 2014, and 2015. (a) Vertical profiles of reef CI‐1 during each scan with average sea levels between each scan period (dashed lines). (b) Mean vertical accretion rates by elevation (solid lines) and mean relative sea levels (dashed horizontal lines) during the scan time steps. Light red and dark blue bracketed bars represent the predicted optimal growth zone (OGZ) based on sea‐level data for relevant scan periods. (c) Reef volume change at each elevation (solid lines) with mean sea‐level data (dashed lines)

With the centennial reef, mean sea levels over the two time periods increased 3.9 cm, from −0.021 m NAVD88 (averaged through June 2012–June 2014) to 0.018 m NAVD88 (averaged through June 2014–June 2015) (Figure 7a). During the first time step (average sea level), the centennial reef only exhibited growth between the elevations of −0.41 to −0.09 m NAVD88, with losses of 2 cm/year on top of the reef (Figure 7b), and maximum growth (2.9 cm/year) at −0.29 m NAVD88 (Table 2). The second time step (high sea level) revealed accretion over the entire elevation gradient as the OGZ shifted higher with maximum growth (4.4 cm/year) at 0.01 m NAVD88. While sea levels and the predicted OGZ raised 4 cm in elevation, the upper growth boundary experienced an increase from −0.09 m to 0.13 m NAVD88 (22 cm). We also did not capture a lower critical boundary where growth dropped near zero, which is likely not resolvable because this reef extended below the water line during low tide. Reef volume gain during the first time step hovered just below 0.1 m^3^/year within the OGZ (Figure 7c). In contrast, the second time step had increasing volume change beginning in the OGZ and peaking (1.0 m^3^/year) just above MSL.

## 4 DISCUSSION

Changes in reef morphology and reef‐wide growth were tightly aligned with month‐to‐year patterns in sea level (Figures 5, 6, 7, Table 2). The magnitude and direction of these interannual fluctuations in sea level coincided with similarly scaled growth and erosion that manifested along reef profiles. When compared to other intertidal or shallow subtidal habitats such as salt marsh and mangrove (Baustian et al., 2012; Bhomia et al., 2015; Cahoon et al., 2006; Perry et al., 2015; Sasmito et al., 2016), surface accretion across all reef generations exceeded the rates of accretion in other coastal biogenic habitats. Additionally, the predicted OGZ and growth ceiling, based on sea level during each scan period, paralleled general growth trends across reefs of all ages, further supporting its use as a management tool in oyster‐reef conservation and restoration.

Variations in water quality did not appear to have a strong influence on reef growth, but fluctuations in salinity may be responsible for nonconformities in the expected growth pattern. Water temperatures over the entire study period did not display dramatic deviations that would explain differences in growth between years (Figure 3c). Overall temperatures varied about a degree Celsius or less between scan periods, having cooler temperatures during the second time steps. Cooler temperatures would be associated with less growth in *C. virginica* (Dame, 1972), which is contrary to our results, indicating that temperatures had little effect on how growth manifested on reefs. In contrast, salinity decreased throughout the entire study period, which could have impacted how growth manifested at lower elevations along the reef profiles. *Crassostrea virginica* is robust to fluctuations in salinity, and the range (15–36 ppt) is not outside of the Eastern oyster's tolerance (Shumway, 1996). Salinities below 25 ppt may actually be more conducive for oyster growth, especially in subtidal waters (Walles et al., 2016) where fresher water may hinder predators (e.g., gastropods) and competitors (e.g., macroalgae), which could explain high growth below the OGZ on the decade‐old reefs after periods of pronounced lower salinity. Both salinity and seasonal temperature cycles can influence oyster reproduction and recruitment, and this can result in interannual variability in larval settlement patterns (Ortega & Sutherland, 1992). While we did not collect oyster spatfall data for this study, other research conducted within our study area and adjacent waters has shown larval availability to be high within the estuaries of North Carolina and that interannual variability of larval supply is relatively low in the more saline sounds (Carroll, Riddle, Woods, & Finelli, 2015; Ortega & Sutherland, 1992; Puckett & Eggleston, 2012). In these waters, the major determinants of oyster recruitment are the postsettlement processes of predation and competition (Carroll et al., 2015; Fodrie et al., 2014).

Our study was not designed as a controlled experiment to control for a suite of abiotic and biotic factors. However, changes to biotic or abiotic influences, other than salinity, should generally impact the magnitude of growth profiles, rather than shifting growth curves upward or downward as we observed. For instance, increased thermal stress or disease should decrease the magnitude of a growth curve overall but not change the elevations associated with growth. The exceptions to this response would be those processes dictated by tidal‐exposure stressors (e.g., desiccation, predation, and competition), which shift correspondingly with sea level, reinforcing sea level as the primary control (and certainly the most parsimonious based on the available data).

### 4.1 Decade‐old reefs

Distinct patterns of growth were exhibited by the decade‐old reefs during the two sampling timeframes (Figure 5) coinciding with shifts in sea level. The initial OGZ occurred in the mid–low intertidal with these reefs showing erosion above −0.07 m. Although the reefs extended above MSL (−0.03 m NAVD88) during the first time step, they predominantly experienced erosion across their plateaus between 2010 and 2012. Erosion was most likely the response of these reefs returning to equilibrium after a year of high water preceding the first scan (Figure 3b). This would have temporarily increased the growth ceiling before waters returned to a lower stand, exposing the reef crest to higher desiccation stress and potentially greater foraging by avian predators (American Oystercatcher, *Haematopus palliates*), resulting in oyster mortality. Thus, it appears that while oysters cement together to create a solid reef matrix, oyster mortality within the taphonomically active zone (layer of living oysters) due to overexposure could compromise the outer reef structure, making it more susceptible to erosional forces. A similar process has been documented on coral reefs during extreme low tides (Anthony & Kerswell, 2007), and it vertically mirrors dieback of marshes in response to long term over inundation creating highly reduced soils (Koch, Mendelssohn, & Mckee, 1990). Even though oysters cement together, death of an oyster can lead to the valves disconnecting as the adductor muscle releases, which can result in loose shell if wave energy eventually works the valves apart. When larger oysters die within the taphonomically active zone, bigger clumps of oysters can be worked loose and displaced if multiple smaller oysters are attached to a large valve. These areas then appear as erosion spots when the reef is mapped again if the space has not been reoccupied by other oysters in that time. While present, loose oyster shell on or around the reef was not quantified nor is it likely to have a high residence time on the reef crest as these areas are subject to the greatest hydrodynamic energy, and shells are often observed scattered across the adjacent sandflat. Therefore, it is difficult to use loose shell as a metric for erosion on these isolated reefs. We could also have witnessed a natural process of compaction within the reef. As multiple generations of oysters continue to build on one another, the structure of the reef matrix likely condenses and fills the empty cavities of once living oysters. During years of average or higher water, this process is likely compensated by oyster growth on the reef surface and therefore only manifests as loss during periods of protracted low water. Some places, such as areas of the Chesapeake Bay, experience shell loss through dissolution in more acidic conditions; however, our study area is not experiencing pronounced acidification, and dissolution is an unlikely source of reef surface erosion.

For the decade‐old reefs, mean sea level increased 3.7 cm between the two time steps with prolonged higher water during the winter of 2014–2015, which corresponds to increased growth at higher elevations on the reef as both the OGZ and growth ceiling shifted upward (Figure 5). Extended high water can benefit a reef by providing increased accommodation space or the space available for deposition that is usually controlled by sea level. This temporary increase in accommodation space is similar to how short (monthly) periods of high water have been shown to positively impact marsh communities (Morris, Kjerfve, & Dean, 1990). While the average maximum growth remained at 2 cm/year, the overall distribution of this growth encompassed nearly the entire elevation range of the reefs between 2012 and 2015. As there was not a drop in sea level during the time period that would have corresponded to growth lower on the reef than the initial OGZ, it remains to be determined why the lower reef elevations exhibited the same amount of accretion. The lower edges of intertidal reefs are generally less consolidated, making it easier to harvest oysters; consequently, the comparable accretion witnessed on these portions of the reefs could be related to decreased harvesting of study area oysters at this time. Lower reef growth could also be a trend linked to reef maturity, as older reefs have been shown to have greater densities of adult oysters at depth (Ridge et al., 2015). It is also possible that prolonged periods of decreased salinity during the 2014–2015 winter could be responsible for some of the deeper‐reef growth witnessed. Brackish water favors subtidal reef growth (Walles et al., 2016) as it inhibits competition and predation. Therefore, the pronounced drops in salinity during the winter and spring of 2014–2015 could have fostered oyster growth in the shallow subtidal.

### 4.2 Young reefs

Elevations of growth maximums on young reefs also paralleled changes in sea levels (Figure 6). Sea levels during 2012 were higher than the initial time step for the decade‐old reefs, which resulted in an elevated OGZ. When sea levels dropped 2 cm in 2013, maximum growth also occurred 2 cm lower, doubling the average accretion rate while also increasing growth along deeper areas of these reefs. Young reefs appear to have the strongest response to sea‐level changes, but this could be a result of our scan‐period resolution isolating narrow time frames with fairly distinct trends in sea level. Areas of the young reefs exhibited growth as high as 8–11 cm/year after their construction in 2011 (Rodriguez et al., 2014). The sustained high average growth across these reefs indicates they will only require 4–6 years to occupy the accommodation space and reach MSL. Volume changes on young reefs are an order of magnitude greater than the decade‐old reefs because of the greater surface area located within the OGZ coupled with a much higher vertical accretion rate. This pattern of growth, two to three times greater than the decade‐old reefs, follows the modeled maturation of other intertidal habitats such as marshes, which experience rapid growth during immaturity that asymptotes at the rate of RSLR at maturity (Allen, 1990; Jennings, Carter, & Orford, 1995).

### 4.3 Centennial reef

Growth changes on the centennial reef over the study period behaved comparably to patterns displayed by the other study reefs (Figure 7). Peak growth during the initial time step occurred at the base of the predicted OGZ. Similar to the decade‐old reefs, the centennial reef experienced erosion at elevations above −0.07 during a period of relatively low water (2013). However, the higher water of 2014–2015 yielded 4 cm/year accretion in previously eroded areas at or above MSL, which manifests as a comparably large increase in volume across the reef plateau. This growth rate (4 cm/year) is equivalent to the 3.9 cm jump in average sea levels over the two time intervals and is also comparable to the rapid growths displayed by the young reefs. Thus, mature reefs not only follow the intertidal oyster‐reef growth paradigm, they also have the capacity to respond just as rapidly to changes in sea level as immature reefs. This would indicate that mature oyster reefs are not confined to nearly asymptotic growth at the rate of RSLR like that of other coastal habitats. Considering the clarity in response of the centennial reef to the relatively confined sea level trend in 2014–2015, it is possible that we could have measured a similar response on the decade‐old reefs if we had isolated a smaller window of time. Instead, the growth response of the decade‐old reefs is diluted across 3 years of fluctuating sea level.

Growth on the centennial reef was measured at higher elevations than the other reefs included in this study. This reef is much larger than the constructed reefs, and we may be witnessing a certain degree of facilitation (Bruno, Stachowicz, & Bertness, 2003) within the oyster population, similar to the Northern Acorn Barnacles of the New England rocky intertidal (Bertness, 1989), due to thermal buffering and reduced desiccation stress. In fact, each time the centennial reef was sampled there remained ponds of water on the reef's plateau during low tide (appear as dark spots on the reef in Figure 2b). Presence of these ponds shows that the reef is fairly nonporous, retaining water at higher elevations throughout a tidal cycle. This would indicate that, while the OGZ magnitude of large natural reefs corresponds to shifts in sea level, the elevations at which the OGZ manifests may behave differently as a reef matures and expands.

As a larger reef spread across a greater exposure gradient, the degree to which the centennial reef would have been subject to forces impacting surface elevation may have been different than the constructed reefs. Having a much larger mass, the reef may be more prone to compaction in years of low water. Its size and proximity to sandy shoreline may have also fostered higher foraging by the American Oystercatcher during years when the reef was more exposed, although this is likely not as significant as desiccation mortality and reef compaction. Alternatively, the centennial reef is in a more sheltered environment and encompasses a much larger population of oysters compared to the constructed reefs and is adjacent to other reefs of similar size. This would provide a greater localized source of oyster larvae, helping maintain recruitment even during years of lower larval supply relative to the smaller, more exposed constructed reefs (O'Beirn, Heffernan, & Walker, 1995, 1996).

### 4.4 Resilience to sea‐level fluctuations

Oyster reefs appear to be in dynamic equilibrium with sea level. Like other intertidal habitats, decadal or longer measurements of mature reef surface elevation changes would show they track RSLR (DeAlteris, 1988). However, unlike other habitats, annual rates of change in reef vertical relief could be ±5 cm depending on relative sea levels. Prolonged shifts in sea level cause different reef elevations to essentially turn off or on, akin to a phenomenon present in coral reefs (Perry & Smithers, 2011) but operating at a much greater magnitude. Rapid coral reef vertical accretion is on the order of 0.5–0.9 cm/year during reef turn‐on (Perry & Smithers, 2011), while oyster reefs can achieve greater than 2 cm/year regardless of maturity. Therefore, oyster reefs, despite being sessile organisms, are well adapted for tracking this particular climate velocity vector as long as the environment remains estuarine. It should be noted that this outstanding vertical accretion has only been measured on intertidal oyster reefs, and subtidal oyster reef accretion may not respond to fluctuations in sea level. Existing in the intertidal zone mediates the impact of biotic interactions and near bottom hypoxia, to which subtidal reefs are exposed (Lenihan, 1999), allowing for growth to be dictated primarily by sea level and aerial exposure regime assuming no disease or degraded water quality.

The ubiquity of this response across oyster‐reef ages is a testament to their resilience to RSLR as well as their utility and longevity for stabilizing shorelines, likely reducing the potential impacts of the coastal squeeze (Pontee, 2013). This work supports the value of using the OGZ for intertidal oyster population management (Ridge et al., 2015), being an effective predictive tool for oyster‐reef growth patterns. Use of the OGZ will prove highly valuable in restoration projects, particularly the implementation of green infrastructure such as living shorelines that incorporate oyster breakwaters. However, there remains a need to measure oyster‐reef lateral expansion and adjacent benthic‐sediment modification processes. These measurements will help establish whether or not oyster reefs will build landward as they track SLR or eventually create reef islands. Similar to wetland upland transgression with RSLR (Kirwan, Walters, Reay, & Carr, 2016), this process will depend on the ability of oysters to expand up the littoral slope (Ridge, Rodriguez, & Fodrie, 2017). Considering the outstanding vertical growth captured in this study, the primary limiting factor would appear to be the rate of transgression (i.e., expansion up slope) at a particular shoreline.

This study presents evidence that intertidal oyster reefs are highly responsive to short‐term fluctuations in local sea level even at maturation. When compared to other coastal habitats and their capacities for RSLR response, oyster reefs are unparalleled in their ability to maintain surface elevation with changing sea level. Greatest recorded rates of surface elevation change in intertidal and shallow subtidal systems such as marshes, mangroves, and corals are below 1–2 cm/year excluding storm‐related allochthonous sedimentation (Baustian et al., 2012; Bhomia et al., 2015; Perry et al., 2015; Sasmito et al., 2016). Overall, this research further solidifies that oyster reefs are resilient habitats that will become increasingly important in estuarine systems with changing sea level.

## CONFLICT OF INTEREST

None declared.

## AUTHOR CONTRIBUTIONS

All authors participated in project conception, data collection and analysis, and the writing and editing of the manuscript.
