# Supplementary material for: Evidence of exceptional oyster‐reef resilience to fluctuations in sea level
Source: Ecol Evol. 2017 Oct 31;7(23):10409–20. doi: 10.1002/ece3.3473 (PMC5723620; doi:10.1002/ece3.3473)
Supplement: Supplementary file 1 [file ECE3-7-10409-s001.docx]

**Appendix Table 1** Statistical comparisons of water quality data between scan time steps for each reef generation.

| Reef Type | Time  Step | Temperature (ºC)^‡^ | Wilcoxon Rank Sums |  | Salinity (ppt)^‡^ | t-Tests (_DF_) |
| --- | --- | --- | --- | --- | --- | --- |
| Decade-old | 1 | 19.7 ± 7.2 | *Z =* 0.68  *p =* 0.50 |  | 31.8 ± 2.2 | ***t_55_ =* -2.5**  ***p =* 0.015** |
|  | 2 | 18.6 ± 7.3 |  |  | **^*^**29.6 ± 4.0 |  |
|  |  |  |  |  |  |  |
| Young | 1 | 19.7 ± 6.6 | *Z =* 0.84  *p* = 0.40 |  | 32.4 ± 2.8 | ***t_23_ =* -2.4**  ***p* = 0.025** |
|  | 2 | 18.2 ± 7.0 |  |  | **^*^**29.7 ± 3.0 |  |
|  |  |  |  |  |  |  |
| Centennial | 1 | 18.8 ± 6.9 | *Z = -0.02*  *p = 0.99* |  | 30.6 ± 2.9 | *t_32_ =* -1.7  *p* = 0.10 |
|  | 2 | 18.5 ± 8.5 |  |  | 27.7 ± 5.6 |  |

^‡^ Mean ± Standard Deviation

^*^ Significant difference (*P <* 0.05) from previous timestep.
